# Supplementary figures and images for: Extra Virgin Olive Oil-Based Formulations: A “Green” Strategy against Chlamydia trachomatis
Source: Int J Mol Sci. 2023 Aug 11;24(16):12701. doi: 10.3390/ijms241612701 (PMC10454370; doi:10.3390/ijms241612701)

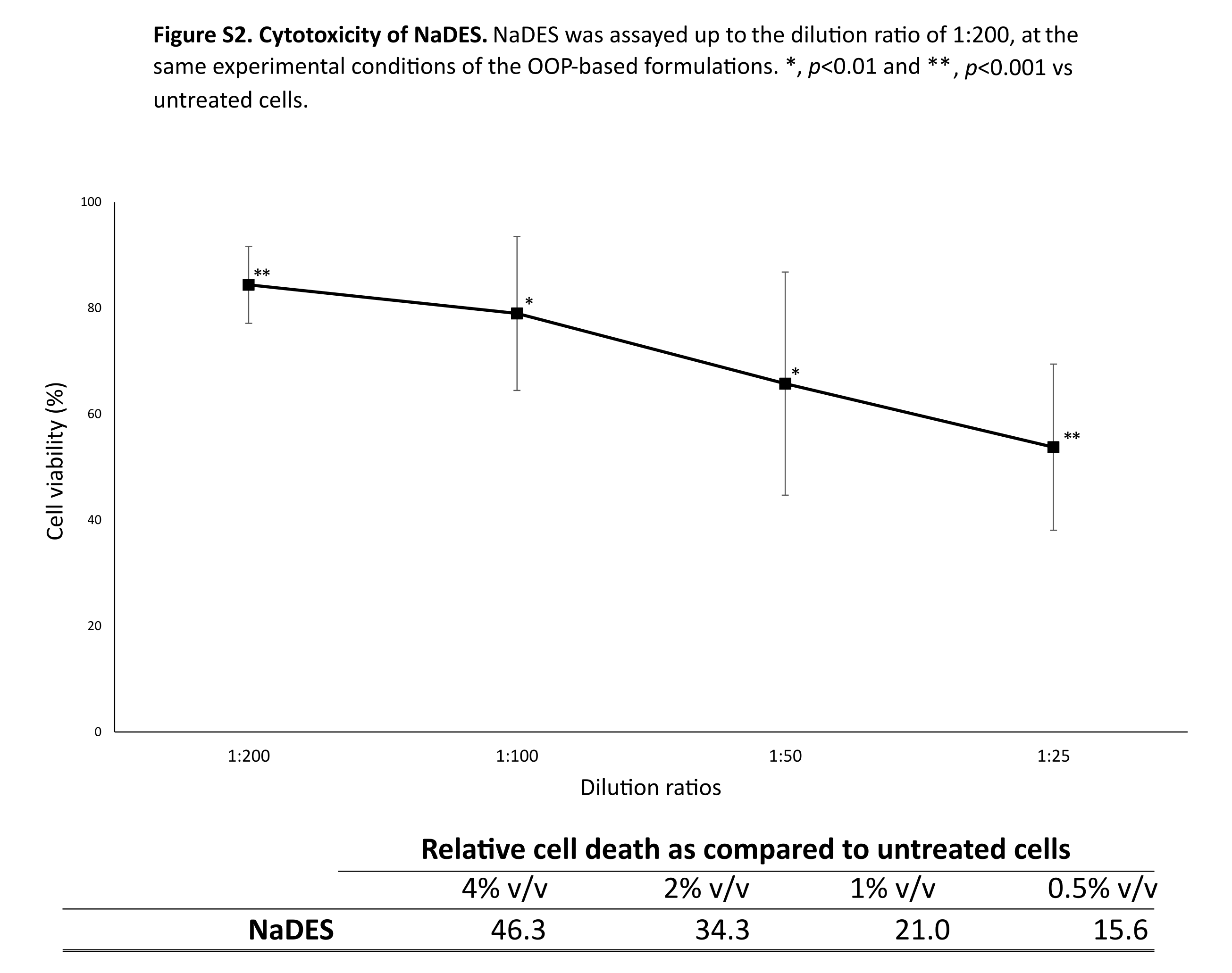

Supplement: Supplementary file 1 [file ijms-24-12701-s001.zip › FigureS2.tiff]

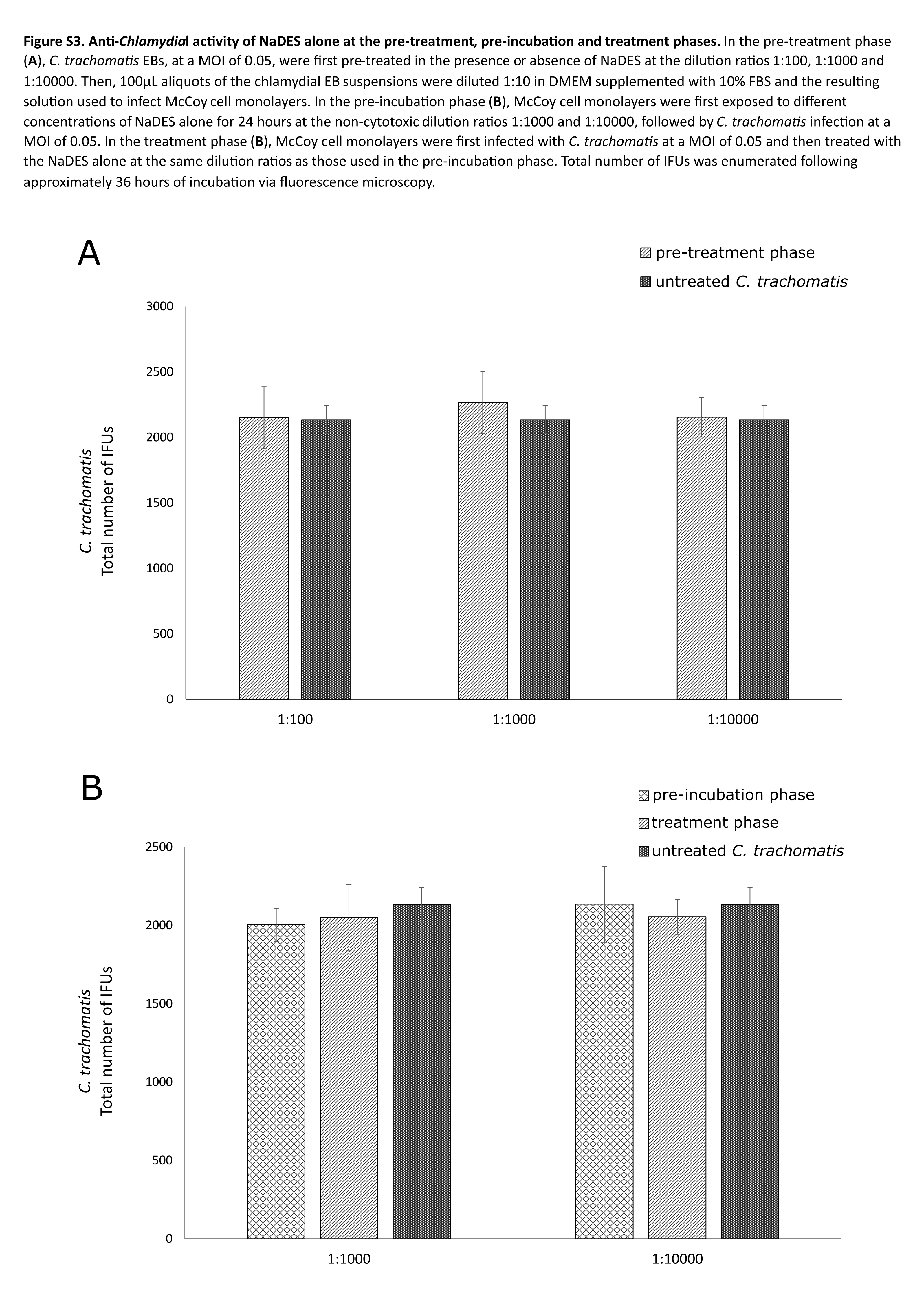

Supplement: Supplementary file 1 [file ijms-24-12701-s001.zip › FigureS3.tiff]
